# Molecular and morphological data supporting phylogenetic reconstruction of the genus *Goniothalamus* (Annonaceae), including a reassessment of previous infrageneric classifications

**DOI:** 10.1016/j.dib.2015.06.021

**Published:** 2015-07-09

**Authors:** Chin Cheung Tang, Daniel C. Thomas, Richard M.K. Saunders

**Affiliations:** a School of Biological Sciences, The University of Hong Kong, Pokfulam Road, Hong Kong, China; b Singapore Botanic Gardens, 1 Cluny Road, Singapore 259569, Singapore

## Abstract

Data is presented in support of a phylogenetic reconstruction of the species-rich early-divergent angiosperm genus *Goniothalamus* (Annonaceae) (Tang et al., Mol. Phylogenetic Evol., 2015) [1], inferred using chloroplast DNA (cpDNA) sequences. The data includes a list of primers for amplification and sequencing for nine cpDNA regions: *atpB-rbcL*, *matK*, *ndhF*, *psbA-trnH*, *psbM-trnD, rbcL*, *trnL-F*, *trnS*-*G*, and *ycf1*, the voucher information and molecular data (GenBank accession numbers) of 67 ingroup *Goniothalamus* accessions and 14 outgroup accessions selected from across the tribe Annoneae, and aligned data matrices for each gene region. We also present our Bayesian phylogenetic reconstructions for *Goniothalamus*, with information on previous infrageneric classifications superimposed to enable an evaluation of monophyly, together with a taxon-character data matrix (with 15 morphological characters scored for 66 *Goniothalamus* species and seven other species from the tribe Annoneae that are shown to be phylogenetically correlated).

**Table:** Specifications table

Subject area	Biology, genetics and genomics
More specific subject area	Phylogenetics
Type of data	Primer sequences, taxon-sequence matrices, sequence alignments, phylogeny, taxon-character matrix
How data was acquired	Primer sequences designed using Primer3, implemented in Geneious v.5.4.3;
	Sequence data generated by PCR and novel sequencing (supplemented with data downloaded from GenBank); phylogeny generated using Bayesian inference methods
	Taxon-character matrix generated following an extensive literature review
Data format	Raw, filtered and analyzed
Experimental factors	n/a
Experimental features	Sequencing of chloroplast DNA and recording of associated morphological characters
Data source location	n/a
Data accessibility	With this article

**Value of the data**

• Data provides a summary of taxa and chloroplast DNA (cpDNA) regions and aligned data matrices that can be used for the phylogenetic reconstruction of *Goniothalamus* (Annonaceae tribe Annoneae) [1].
• Data provides a summary of morphological characters relevant to species in the tribe Annoneae that are important for broader morphological evolutionary studies.
• Comparisons between the resultant phylogeny for *Goniothalamus* species with previous infrageneric classifications [2,3] enable an assessment of congruence between the phylogeny and the infrageneric classifications.

## 1 Data, experimental design, materials and methods

### 1.1 Primer design and summary

Available sequences of nine chloroplast DNA (cpDNA) regions: *atpB-rbcL*, *matK*, *ndhF*, *psbA-trnH*, *psbM-trnD, rbcL*, *trnL-F*, *trnS*-*G*, and *ycf1* were downloaded from GenBank (https://www.ncbi.nlm.nih.gov/genbank/) for species of *Goniothalamus* and related species from Annonaceae tribe Annoneae. Alignment of each region was performed using MAFFT v.7.029b [4] with default settings and the automatic algorithm option. Each alignment was opened in Geneious v.5.4.3 [5] and “Design New Primer” analysis performed with the “Target Region” set as 300–400 bp and other settings kept as default using Primer3 [6,7]. The summary of primer sequences obtained from the analysis and from previous studies [8–18] are listed in Table 1.

### 1.2 DNA sequencing and upload to GenBank

A modified cetyl trimethyl ammonium bromide (CTAB) method [17,20,21] was used for whole genomic DNA. The extracted DNA was amplified using polymerase chain reaction (PCR). 6.4 μl ddH_2_O, 1.5 μl MgCl_2_ (25 mM), 0.25 μl dNTPs (10 mM), 0.375 μl of each forward and reverse primer (10 μM each, listed in Table 1), 0.5 μl bovine serum albumin (BSA, 10 mg/ml), 0.1 μl Flexi-taq DNA polymerase (Promega, Madison, Wisconsin, U.S.A.), and 0.5 μl DNA template were added for each reaction. The following PCR protocol was adopted: 5 min template denaturation at 95 °C followed by 38 cycles of denaturation at 95 °C for 1 min; primer annealing at 50 °C for 1 min; primer extension at 65 °C for 4 min; with the final extension set to 65 °C for 5 min. PCR products were purified, amplified and sequenced by BGI (Hong Kong, PR China) using the BigDye Terminator Cycle Sequencing Kit (Applied Biosystems, Foster City, California, U.S.A.), with sequencing run on an AB 3730 DNA Analyzer (Applied Biosystems). The sequences were uploaded to GenBank (https://www.ncbi.nlm.nih.gov/genbank/). The summary of the taxon-sequence matrix showing the voucher information and molecular data (GenBank accession numbers) of 67 *Goniothalamus* accessions and 14 accessions in the tribe Annoneae of the family Annonaceae for the nine cpDNA regions is presented in Table 2.

### 1.3 Bayesian phylogenetic reconstructions for *Goniothalamus*

The sequences of the taxa listed in Table 2 were downloaded and aligned using MAFFT v.7.029b [4] with default settings and the automatic algorithm option. For manual editing and optimizing, an 11-bp inversion in *psbA-trnH* and a 16-bp region in *ycf1* were excluded from the matrix in Geneious. The aligned and edited matrices of each region are presented as Supplementary material (Alignments 1–9, representing *atpB-rbcL*, *matK*, *ndhF*, *psbA-trnH*, *psbM-trnD, rbcL*, *trnL-F*, *trnS*-*G*, and *ycf1*).

**Fig. 1:**
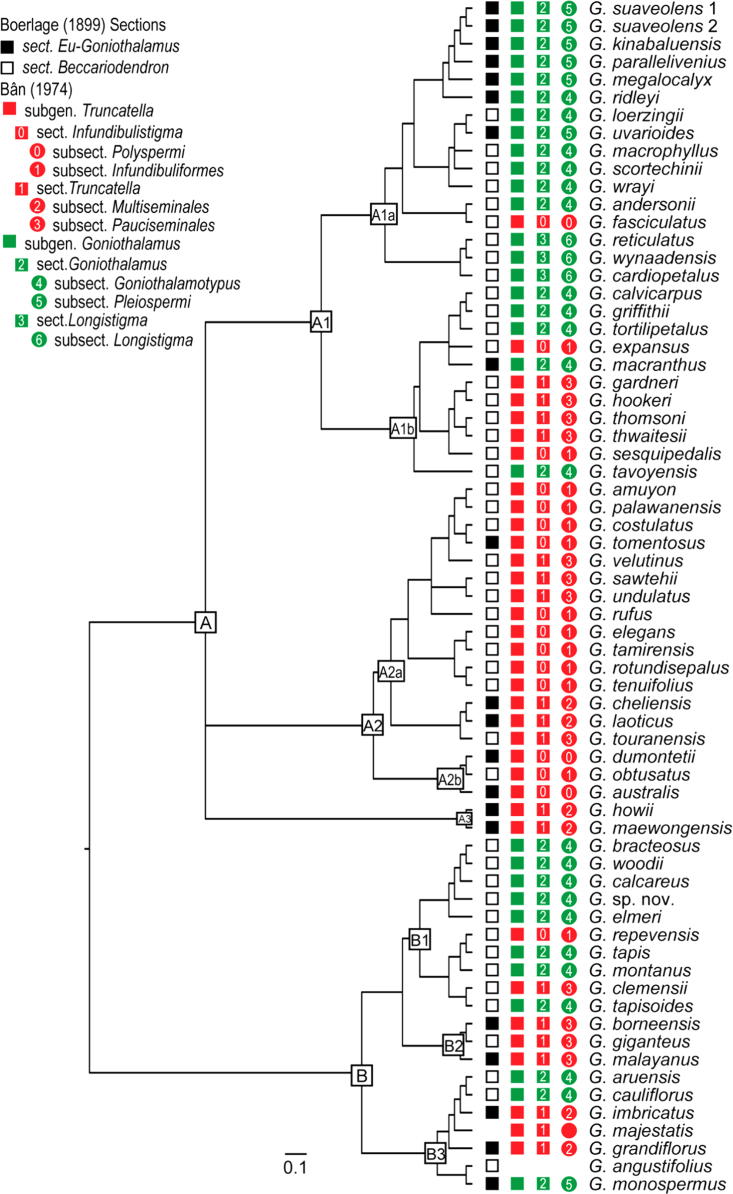
Bayesian 50% majority-rule consensus tree of *Goniothalamus* species, generated from 9-partitioned dataset with all outgroups removed. Previous infrageneric classifications [2,3], published prior to the availability of molecular phylogenetic methods, are superimposed. Boerlage [2] recognized two sections, *Eu-Goniothalamus* (equivalent to the autonymic sect. *Goniothalamus*) and *Beccariodendron*, based on differences in ovule number per carpel. Bân [3] subsequently recognized two subgenera, *Goniothalamus* and *Truncatella*, based on differences in staminal connective shape; each of these subgenera were further divided into sections based on stigma and pseudostyle shape, and subsections based on the number of ovules per carpel. Branch length is proportional to the substitutions rate. Scale bar: 0.1 substitutions per site.

For Bayesian phylogenetic reconstructions, MrBayes v.3.1.2 [22,23] was performed using the online portal in the CIPRES Science Gateway [24]. Data was partitioned according to DNA region identity. The best-fitting evolutionary models were selected using MrModeltest v.2.3 [25] under the Akaike Information Criterion (AIC [26]): GTR+Γ+I was selected for the *psbA-trnH, psbM-F, rbcL*, and *ycf1* partitions; GTR+Γ was selected for the *matK*, *ndhF*, *trnL-F*, and *trnS*-*G* partitions; and the Hasegawa–Kishino–Yano Model with among-site rate variation modeled with a gamma distribution (HKY+Γ) for the *atpB-rbcL* partition. Four independent MCMCMC analyses were run in the Bayesian phylogenetic reconstructions, each with 5,000,000 generations, sampled every 1000th generation. Each run involved three incrementally heated and one cold Markov chain with a temperature parameter of 0.16. The parameters for substitution rates of nucleotide substitution models, character state frequencies and rate variation among sites were unlinked. In order to reduce the likelihood of stochastic entrapment in local tree length optima [27,28], the mean branch length prior was adjusted to 0.01 (brlenspr=unconstrained:exponential (100.0)); all other priors were kept as default. Convergence was assessed by checking that the standard deviation of split frequencies was <0.005. Adequate effective sample sizes (ESS >200) were checked in Tracer v.1.5 [29], which also showed whether the parameter samples were drawn from a unimodal and stationary distribution. The “Cumulative” and “Compare” functions of AWTY [30] were used to evaluate stationarity of posterior probabilities of splits within runs and convergence between different runs. 25% burn-in of initial samples of each run was excluded and a 50% majority-rule consensus tree (see Interactive Phylogenetic Tree 1) was calculated from the post-burn-in trees. A phylogeny with 66 *Goniothalamus* species was extracted from the resultant 50% majority-rule consensus tree. Previous infrageneric classifications [2,3] are superimposed onto the phylogeny to show congruence (Fig. 1).

### 1.4 Taxon-character data matrix

Morphological characters including vegetative, floral, fruit and seed characters were assessed from living and herbarium material (BRUN, HKU, K, L, NY and US herbaria). A total of 14 vegetative, floral, fruit and seed characters were assessed from living and herbarium material, supplemented by species descriptions [31–53]. A summary of 14 characters of 66 *Goniothalamus* species and seven species in the tribe Annoneae are shown in Supplementary Table 1.

## Figures and Tables

**Table 1:** List of primers used for amplification and sequencing of nine DNA regions.

Region	Primer	Sequence (5′–3′)	Source
*atpB-rbcL*	*atpB-rbcL*-2	CCAACACTTGCTTTAGTCTCTG	[14]
	*atpB-rbcL*-c1b	TGGATGAATTMTGGCCATTTTCACA	[1]; this study
	*atpB-rbcL*-c2a	TGGCGCAACCCAATCTTGTT	[1]; this study
	*atpB-rbcL*-c2b	AGTCGCGAGGAGGTTTTTCA	[1]; this study
	*atpB-rbcL*-c3a	GGATGCTGAAATAAAGAACAACAGCCA	[1]; this study
	*atpB-rbcL*-c3b	ACGTCCAATAGCARGTTAATCGGT	[1]; this study
	*atpB-rbcL*-c4a	TGGTGCCAACGAAATCAACCGCW	[1]; this study
	*atpB-rbcL*-3	AGTGTGGAAACCCCAGGATCAGAAG	[10]
*matK*	*matK*-1a	TAATACCTCACCCCGTCCATCTGG	Designed by Y.C.F. Su
	*matK*-c1b	TGTGTTCGCTCGAGAACAGTTCCA	[1]; this study
	*matK*-c2a	CCGTTTGTTCAAAAGAGAATCGGA	[1]; this study
	*matK*-11b	RATCCTGTCCGGTTGAGACCACAA	Designed by Y.C.F. Su
	*matK*-449F	AGAAATGGAAATCTTACCTTGTCC	[17]
	*matK*-824R	ATCCGCCCAAATYGATTGATAATA	[17]
*ndhF*	*ndhF*-1F	ATGGAACAKACATATSAATATGC	[9]
	*ndhF*-c1bR	CCTAAGATTCCTAATAATAAACCA	[1]; this study
	*ndhF*-c2aF	TGGGAACTAGTGGGAATGTGCTCGT	[1]; this study
	*ndhF*-689R	GGCATCRGGYAACCATACATGAAG	[16]
	*ndhF*-c1bF	TGGTTTATTATTAGGAATCTTAGG	[1]; this study
	*ndhF*-c3bR	GCAGCTCGATAAGAACCTATACCTRG	[1]; this study
	*ndhF*-972F	GTCTCAATTGGGTTATATGATG	[9]
	*ndhF*-c4bR	AYCCTRCCGCRGAAYAAGCT	[1]; this study
	*ndhF*-c5aF	TGTGGTATTCCGCCCCTTGCT	[1]; this study
	*ndhF*-c5bR	TGTCYGACTCATGGGGRTATGYRG	[1]; this study
	*ndhF*-LBCF	TCAATAYCTATATGGGGGAAAG	[16]
	*ndhF*-c6bR	ATTGGTGGGGTTAAYARTTTYGAY	[1]; this study
	*ndhF*-c5bF	CYRCATAYCCCCATGAGTCRGACA	[1]; this study
	*ndhF*-2210R	CCCCCTAYATATTTGATACCTTCTCC	[9]
*psbA-trnH*	*psbA*	GTTATGCATGAACGTAATGCTC	[19]
	*psbAtrnH*-c1b	TCGACCATGAACYCGYCARA	[1]; this study
	*psbAtrnH*-c2a	GTTGTTGAAGGATCAGRTCAATGCCA	[1]; this study
	*trnH*_(ham-GUG)_	CGCGCATGGTGGATTCACAATCC	[13]
*psbM-trnD*	*psbM*-*F*	AGCAATAAATGCRAGAATATTTACTTCCAT	[15]
	*psbM*-c1a	TTCGGGATCTAATCCCATAGAAAWACT	[1]; this study
	*psbM*-c2a	TYSRATCAGGAATCYCGTGG	[1]; this study
	*psbM*-c1b	TGGAYCTGTGACCGATGTAAGACCG	[1]; this study
	*psbM*-c3a	CCCTCGAAAGARRKRGGGCGK	[1]; this study
	*psbM*-c2b	TCCAAGGAAGGAGGATACTGACCA	[1]; this study
	*psbM*-c4a	ACTCTGTCGCCGCCGAGATAAC	[1]; this study
	*psbM*-c3b	AGARAGTGCCCATATGTTTTCCG	[1]; this study
	*psbM*-c5a	AGGYGATACCAYCGCTCAATCC	[1]; this study
	*psbM*-c4b1	AGGAGGGACAAGARGCAGGGC	[1]; this study
	*psbM*-c4b2	TTCGAGCCCCGTCAGTCCCG	[1]; this study
	*trnD*_(GUC)_-R	GGGATTGTAGYTCAATTGGT	[15]
*rbcL*	*rbcL*-7F	GATTCAAAGCTGGTGTTAAAGAGT	[17]
	*rbcL*-c1b	GGAATTCGCAAGTCYTCTAGGCGT	[1]; this study
	*rbcL*-c2a	TCGAGCCTGTTGCTGGAGAGGA	[1]; this study
	*rbcL*-724R	TCGCATGTACCTGCAGTAGC	[11]
	*rbcL*-c3a	CGCCAAGAACTACGGTAGRGCG	[1]; this study
	*rbcL*-c3b	TCCCGTTCCCCCTCCAGTTT	[1]; this study
	*rbcL*-4a	GAGACAACGGCCTRCTTCTTCACA	Designed by Y.C.F. Su
	*rbcL*-5a	ATCGCGCAATGCATGCAGTTAT	Designed by Y.C.F. Su
	*rbcL*-5b	ACGTCCCTCATTCCGAGCTTGTA	Designed by Y.C.F. Su
	*rbcL*-c7a	TCGGCGGAGGAACTTTAGGACA	[1]; this study
	*rbcL*-1381R	TCGAATTCGAATTTGATCTCCTTC	[17]
*trnS-G*	*trnS*_(GCU)_	GCCGCTTTAGTCCACTCAGC	[12]
	*trnSG-c1b*	ASYGTTCAAACAAAGTTTTKATCACGA	[1]; this study
	*trnSG-c2a*	TCYATTCCTAYGACAYTCACTCCTGT	[1]; this study
	*trnSG-c2b*	TCGTTACTGAAGTTCCGKCTCG	[1]; this study
	*trnSG-c3a*	CGGATTCTTGTACAACTCATTCTTCTG	[1]; this study
	*trnG*_(UCC)_	GAACGAATCACACTTTTACCAC	[12]
*trnL-F*	*trnLF -13F*	GACGCTACGGACTTGATTGGATT	[17]
	*trnLF-c1b*	TGACATGTAGAACGGGACTCTCTCT	[1]; this study
	*trnLF-c2a*	ACGTATACATAYCGTAGCATCAAACG	[1]; this study
	*trnLF-c2b*	AYTCCTTGCCCATTCATTATCTGTTCA	[1]; this study
	*trnLF-e*	GGTTCAAGTCCCTCTATCCC	[8]
	*trnLF-960R*	AGCTATCCCGACCATTCTC	[17]
*ycf1*	*ycf1*-M935F	AGAACAGTCGGACCAAAAGA	[18]
	*ycf1*-M1792R	TGACATACTGAAACGACTGCC	[18]

**Table 2:** Summary of voucher information and GenBank accession numbers of the 81 accessions.

Voucher information				GenBank accession numbers								
Taxon name	Origin	Voucher	Collection date	*atpB-rbcL*	*matK*	*ndhF*	*psbA-trnH*	*trnL-F*	*trnS-G*	*ycf1*	*rbcL*	*psbM-trnD*
*Annona dumetorum* R.E.Fr.	Dominican Republic	*Abbott, J.R. 20966* (FLAS)	6 June 2006	–	GQ139704	–	EU420856	EU420838	–	GU937352	EU420856	–
*Annona glabra* L.	USA	*Chatrou, L.W. 467* (U)		EF179246	GQ139717	EF179281	AY841596	AY841673	EF179323	GU937365	AY841596	–
*Annona herzogii* (R.E.Fr.) H.Rainer	Bolivia	*Chatrou, L.W. et al. 347* (U)		EF179273	DQ125062	EF179308	AY841656	AY841734	EF179350	–	AY841656	–
*Annona mucosa* Jacq.		*Abbott, J.R. 21032* (FLAS)		–	GQ139705	–	EU420870	EU420852	–	GU937353	EU420870	–
*Annona muricata* L.		*Chatrou, L.W. 468* (U)		EF179247	AF543722	EF179282	AY743440	AY743459	EF179324	–	AY743440	–
*Annona reticulata* Sieber ex A.DC.	Bolivia	*Chatrou, L.W. et al. 290* (U)		–	JQ586491	–	EU420863	EU420845	–	–	EU420863	–
*Annona squamosa* L.		*Nakkuntod, M. 45* (BCU)		–	EU715064	–	EU420865	EU420847	–	–	EU420865	–
*Anonidium* sp. Cheek 7896	Cameroon	*Cheek, M. 7896* (K)		EF179248	DQ125051	EF179283	AY841598	AY841675	EF179325	–	AY841598	–
*Asimina longifolia* Kral	USA	*Weerasooriya, A.D. s.n.* (MISS)		EF179251	GQ139707	EF179286	DQ124939	GQ139885	EF179328	GU937355	DQ124939	–
*Asimina rugelii* B.L.Rob.		*Abbott, J.R. 22361* (FLAS)		–	GQ139706	–	JQ513887	GQ139881	–	GU937354	JQ513887	–
*Asimina triloba* Dunal		*Chatrou, L.W. et al. 276* (U)		EF179252	GQ139711	AY218171	AY743441	AY743460	EF179329	GU937349	AY743441	–
*Disepalum platypetalum* Merr.		*Takeuchi, W. & Sambas 18201*		EF179257	DQ125057	EF179292	–	–	EF179334	–	–	–
*Disepalum pulchrum* (King) J.Sinclair		*Chan, R. 192* (FLAS)		–	GQ139736	–	JQ513888	GQ139909	–	GU937383	JQ513888	–
*Goniothalamus tapis* Miq.	Thailand	*Keßler, P.J.A. 3193* (L)		EF179262	DQ125058	EF179297	AY841622	AY841700	EF179339	–	AY841622	–
*Goniothalamus amuyon* Merr.	Philippines	*Tang, C.C. 20100907* (HKU)	7 Sept 2010	KM818518	KM818567	KM818648	KM818728	KM818898	KM818916	KM818979	KM818839	KM818755
*Goniothalamus andersonii* J.Sinclair	Borneo	*Anderson, J.A.R. S12596* (L)	18 May 1961	KM818519	KM818568	–	KM818711	KM818867	KM818949	–	KM818789	–
*Goniothalamus angustifolius* (A.C.Sm.) B.Xue & R.M.K. Saunders	Fiji	*Gillespie, J.W. 2198* (A)	9 Aug 1927	–	KM818569	KM818632	KM818732	KM818878	KM818937	KM818983	KM818797	–
*Goniothalamus aruensis* Scheff.	New Guinea	*Regalado, J. & Takeuchi, W. 1409* (L)	26 Jun 1995	KM818520	KM818570	KM818640	KM818706	KM818868	KM818918	–	KM818791	–
*Goniothalamus australis* Jessup	Australia	*Unknown collector 3178* (HKU)	17 Jun 2009	KM818521	KM818571	KM818638	KM818709	KM818887	KM818910	KM818973	KM818836	KM818769
*Goniothalamus borneensis* Mat-Salleh	Borneo	*Arbainsyah et al. AA1011* (L)	21 Feb 1995	KM818522	KM818572	KM818673	–	KM818893	KM818952	–	KM818826	KM818747
*Goniothalamus bracteosus* Bân	Borneo	*Clemens, J. & Clemens, M.S. 27619* (L)	17 Dec 1931	–	KM818573	–	KM818730	KM818906	KM818967	–	KM818796	–
*Goniothalamus calcareus* Mat-Salleh	Borneo	*Ahmad Ali, J. BRUN23929* (BRUN)	10 July 2012	–	–	–	KM818717	–	KM818927	KM818994	KM818810	–
*Goniothalamus calvicarpus* Craib	Cultivated	*Saunders, R.M.K., Su, Y.C.F. & Chalermglin, P. 04/13* (HKU)	25 Jul 2004	KM818523	KM818574	KM818647	KM818702	KM818874	KM818934	KM819005	KM818809	KM818775
*Goniothalamus cardiopetalus* Hook.f. & Thomson	India	*Raghavan, R.S. 86311* (L)	16 Feb 1963	KM818524	KM818575	KM818654	KM818692	KM818879	KM818912	–	KM818799	KM818752
*Goniothalamus cauliflorus* K.Schum.	Papua New Guinea	*Hartley, T.G. 9911* (L)	15 Feb 1962	KM818525	KM818576	KM818663	KM818696	KM818869	KM818919	–	KM818807	KM818757
*Goniothalamus cheliensis* Hu	Cultivated	*Saunders, R.M.K., Su, Y.C.F. & Chalermglin, P. 04/22* (HKU)	25 Jul 2004	KM818526	KM818577	KM818661	KM818678	KM818901	KM818926	KM818992	KM818831	KM818758
*Goniothalamus clemensii* Bân	Borneo	*Beaman, J.H. 8184* (L)	3 Jan 1984	–	KM818578	–	KM818736	KM818844	KM818915	–	KM818780	–
*Goniothalamus costulatus* Miq.	Java	*Martati, T. 169* (L)	15 Sep 1960	–	KM818579	–	KM818737	KM818865	KM818945	–	KM818805	–
*Goniothalamus dumontetii* R.M.K. Saunders & Munzinger	New Caledonia	*Dumontet, V. & Poullain, C. 716* (HKU)	15 Jun 2006	–	KM818580	–	KM818729	KM818861	KM818954	–	KM818840	–
*Goniothalamus elegans* Ast	Thailand	*Nakkuntod, M. 40* (BCU)	28 Oct 2005	KM818527	KM818581	KM818676	KM818707	KM818850	KM818955	KM818997	KM818817	–
*Goniothalamus elmeri* Merr.	Philippines	*Rosario et al. 11-014* (University of Santo Tomas Herbarium)	s.a.	–	KM818582	KM818639	KM818677	KM818882	KM818924	KM819003	KM818811	–
*Goniothalamus expansus* Craib	Thailand	*Kitamura, S. MN22* (BCU)	9 Jun 2004	–	KM818583	KM818634	KM818714	KM818853	KM818931	KM818987	KM818829	–
*Goniothalamus fasciculatus* Boerl.	Borneo	*Keßler, P.J.A. et al. 2846* (HKU)	10 Apr 2000	KM818528	KM818584	KM818636	–	KM818890	KM818950	–	–	–
*Goniothalamus gardneri* Hook.f. & Thomson	Sri Lanka	*Tillekaratne, H.I. G29* (HKU)	s.a.	KM818529	KM818585	KM818656	KM818704	KM818871	KM818923	KM819001	KM818784	KM818773
*Goniothalamus giganteus* Hook.f. & Thomson	Cultivated	*Saunders, R.M.K., Su, Y.C.F. & Chalermglin, P. 04/28* (HKU)	25 Jul 2004	KM818530	KM818586	KM818655	KM818698	KM818892	KM818963	KM818996	KM818837	KM818754
*Goniothalamus grandiflorus* Boerl.	Papua New Guinea	*Takeuchi, W.N. 8771* (L)	11 Feb 1993	KM818531	KM818587	KM818637	KM818691	KM818851	KM818930	–	KM818802	KM818770
*Goniothalamus griffithii* Hook.f. & Thomson	Thailand	*Saunders, R.M.K. & Chalermglin, P. 04/30* (HKU)	28 Jul 2004	KM818532	KM818588	KM818651	KM818701	KM818894	KM818939	KM819000	KM818798	KM818748
*Goniothalamus hookeri* Thwaites	Sri Lanka	*Ratnayake, R.M.C.S. 100* (HKU)	10 Feb 2003	KM818533	KM818589	KM818657	KM818734	KM818872	KM818956	–	KM818814	KM818774
*Goniothalamus howii* Merr. & Chun	China	*Wang, X.B. W2011003* (HUTB)	3 Aug 2011	KM818534	KM818590	–	KM818689	KM818886	KM818938	KM818986	KM818833	KM818767
*Goniothalamus imbricatus* Scheff.	Papua New Guinea	*Bau, B. LAE89112* (LAE)	s.a.	KM818535	KM818591	–	KM818722	KM818847	KM818946	KM818998	KM818806	KM818753
*Goniothalamus kinabaluensis* Bân ex Mat-Salleh	Borneo	*Vogel, E.F. de 8387* (L)	18 Oct 1986	KM818536	KM818592	KM818672	KM818684	KM818876	KM818935	–	KM818787	KM818745
*Goniothalamus laoticus* (Finet & Gagnep.) Bân	Cultivated	*Saunders, R.M.K., Su, Y.C.F. & Chalermglin, P. 04/9* (HKU)	25 Jul 2004	KM818537	KM818593	KM818666	KM818699	KM818881	KM818959	KM818993	KM818808	KM818760
*Goniothalamus loerzingii* R.M.K. Saunders	Sumatra	*Kostermans, A.J.G.H. 22015* (L)	13 Dec 1965	–	KM818594	–	KM818724	KM818902	KM818947	–	KM818782	–
*Goniothalamus macranthus* Boerl.	Andamans	*King׳s collector 347* (L)	1884	KM818538	KM818595	KM818643	KM818695	KM818873	KM818928	KM818995	KM818792	KM818776
*Goniothalamus macrophyllus* (Blume) Hook.f. & Thoms.	Cultivated	*Saunders, R.M.K., Su, Y.C.F. & Chalermglin, P. 04/16* (HKU)	25 Jul 2004	KM818539	KM818596	KM818665	KM818688	KM818897	KM818940	KM819002	KM818843	KM818766
*Goniothalamus maewongensis* R.M.K. Saunders & Chalermglin	Thailand	*Saunders, R.M.K., Nakkuntod, M. & Chalermglin, P. 04/35* (HKU)	29 Jul 2004	KM818540	KM818597	KM818659	KM818725	KM818888	KM818962	KM818977	KM818838	KM818746
*Goniothalamus majestatis* Kessler	Sulawesi	*McDonald, J.A. 3896* (L)	26 July 1993	KM818541	KM818598	–	KM818713	KM818903	KM818958	–	KM818788	KM818756
*Goniothalamus malayanus* Hook.f. & Thomson	Cultivated	*Saunders, R.M.K., Su, Y.C.F. & Chalermglin, P. 04/24* (HKU)	25 Jul 2004	KM818542	KM818599	KM818650	KM818718	KM818891	KM818914	KM819006	KM818835	KM818743
*Goniothalamus megalocalyx* I.M.Turner & R.M.K. Saunders	Borneo	*Tang, C.C. et al. TCC117* (HKU)	11 Nov 2011	KM818543	KM818600	KM818645	KM818726	KM818885	KM818960	KM819007	KM818822	KM818763
*Goniothalamus monospermus* (A.Gray) R.M.K. Saunders	Fiji	*Smith, A.C. 5111* (L)	7 Jul-18 Sep 1947	–	KM818601	–	KM818735	–	KM818969	–	KM818790	–
*Goniothalamus montanus* J.Sinclair	Peninsular Malaysia	*Soepadmo, E. & Suhaimi, M. 43* (L)	11 Nov 1989	KM818544	KM818602	KM818674	KM818710	KM818856	KM818932	–	KM818813	–
*Goniothalamus obtusatus* (Baill.) R.M.K. Saunders	New Caledonia	*Veillon, J.M. 7591* (NOU)	25 Nov 1992	KM818545	KM818603	KM818660	KM818687	KM818883	KM818911	KM818981	KM818815	–
*Goniothalamus palawanensis* C.C. Tang & R.M.K. Saunders	Philippines	*Tang, C.C. TCC12* (HKU)	31 May 2012	–	KM818604	–	KM818716	KM818855	KM818925	KM818976	KM818793	–
*Goniothalamus parallelivenius* Ridl.	Borneo	*Tang, C.C. et al. TCC50* (HKU)	16 May 2011	KM818546	KM818605	KM818635	KM818683	KM818880	KM818941	–	KM818801	KM818765
*Goniothalamus repevensis* Pierre ex Finet & Gagnep.	Cultivated	*Saunders, R.M.K., Su, Y.C.F. & Chalermglin, P. 04/8* (HKU)	25 Jul 2004	KM818547	KM818606	KM818664	KM818723	KM818877	KM818936	–	KM818795	KM818749
*Goniothalamus reticulatus* Thwaites	Sri Lanka	*Saunders, R.M.K. & Weerasooriya, A.D. 00/7* (HKU)	17 Jun 2000	KM818548	KM818607	–	–	–	KM818913	–	KM818786	KM818742
*Goniothalamus ridleyi* King	Peninsular Malaysia	*Soepadmo, E. & Suhaimi, M. 341* (L)	16 Feb 1991	KM818549	KM818608	–	KM818739	KM818860	KM818951	KM818985	KM818830	–
*Goniothalamus rotundisepalus* M.R.Hend.	Thailand	*Larsen, K. & Larsen, S.S. 32826* (AAU)	2 Mar 1974	KM818550	KM818609	KM818649	KM818693	KM818857	KM818908	–	KM818794	KM818759
*Goniothalamus rufus* Miq.	Borneo	*Keßler, P.J.A. et al. 2482* (L)	10 Mar 1999	KM818551	KM818610	–	KM818727	KM818848	KM818943	–	KM818819	–
*Goniothalamus sawtehii* C.E.C.Fisch.	Cultivated	*Saunders, R.M.K., Su, Y.C.F. & Chalermglin, P. 04/14* (HKU)	25 Jul 2004	KM818552	KM818611	KM818646	KM818680	KM818895	KM818942	KM819004	KM818785	KM818751
*Goniothalamus scortechinii* King	Peninsular Malaysia	*Noorsiha, A. et al. FRI 39427* (L)	21 Sep 1993	KM818553	KM818612	KM818670	KM818712	KM818845	KM818929	KM818988	KM818781	KM818744
*Goniothalamus sesquipedalis* Hook.f. & Thomson	India	*Griffith, W. s.n. [= Herb. E. India Co. 402A]* (L)	*s.a.*	KM818554	KM818613	KM818667	KM818719	KM818904	KM818907	KM818984	KM818825	KM818740
*Goniothalamus* sp. nov. tcc10	Philippines	*Tang, C.C. TCC10* (HKU)	31 May 2012	–	KM818614	KM818675	KM818715	KM818864	KM818944	KM818980	KM818821	–
*Goniothalamus suaveolens* 1 Becc.	Borneo	*Tang, C.C. TCC32* (HKU)	10 May 2011	KM818555	KM818616	–	KM818682	KM818858	KM818933	KM818982	KM818800	KM818762
*Goniothalamus suaveolens* 2 Becc.	Borneo	*Atkins, S. 466* (L)	14 Jul 1993	–	KM818615	–	KM818681	KM818884	KM818968	KM818999	KM818818	–
*Goniothalamus tamirensis* Pierre ex Finet & Gagnep.	Cultivated	*Saunders, R.M.K., Su, Y.C.F. & Chalermglin, P. 04/23* (HKU)	25 Jul 2004	KM818556	KM818617	KM818662	KM818700	KM818866	KM818917	KM818990	KM818832	KM818761
*Goniothalamus tapisoides* Mat-Salleh	Borneo	*Tang, C.C. et al. TCC51* (HKU)	16 May 2011	KM818557	KM818618	KM818641	KM818686	KM818899	KM818920	–	KM818823	KM818771
*Goniothalamus tavoyensis* Chatterjee	Cultivated	*Saunders, R.M.K., Su, Y.C.F. & Chalermglin, P. 04/11* (HKU)	25 Jul 2004	KM818558	KM818619	KM818633	KM818690	KM818854	KM818961	–	KM818841	KM818750
*Goniothalamus tenuifolius* King	Cultivated	*Saunders, R.M.K., Su, Y.C.F. & Chalermglin, P. 04/17* (HKU)	25 Jul 2004	KM818559	KM818620	KM818669	KM818694	KM818889	KM818909	KM818974	KM818842	KM818741
*Goniothalamus thomsoni* Thwaites	Sri Lanka	*Kostermans, A.J.G.H. 25485* (L)	31 Aug 1974	–	KM818621	–	KM818733	KM818875	KM818971	–	KM818834	–
*Goniothalamus thwaitesii* Hook.f. & Thomson	India	*Beddome, R.H. 299* (PDA)	*s.a.*	KM818560	KM818622	KM818653	KM818703	KM818849	KM818922	–	–	KM818772
*Goniothalamus tomentosus* R.M.K. Saunders	Peninsular Malaysia	*Whitmore, T.C. FRI 3851* (L)	21 May 1967	KM818561	KM818623	–	KM818738	KM818846	KM818964	–	KM818783	–
*Goniothalamus tortilipetalus* M.R.Hend.	Thailand	*Nakkuntod, S. 58* (HKU)	25 Nov 2005	–	KM818624	KM818642	KM818708	KM818905	KM818948	–	KM818828	–
*Goniothalamus touranensis* Ast	Indochina	*Clemens, J. & Clemens, M.S. 4187* (NY)	May-Jul 1927	–	KM818625	–	KM818731	KM818870	KM818965	–	KM818804	–
*Goniothalamus undulatus* Ridl.	Cultivated	*Saunders, R.M.K., Su, Y.C.F. & Chalermglin, P. 04/25* (HKU)	25 Jul 2004	KM818562	KM818626	KM818652	KM818679	KM818896	KM818921	KM818978	KM818820	KM818777
*Goniothalamus uvarioides* King	Peninsular Malaysia	*Kochummen, K.M. FRI 2344* (L)	24 May 1967	–	KM818627	KM818658	KM818685	KM818852	KM818966	KM818975	KM818827	–
*Goniothalamus velutinus* Airy Shaw	Borneo	*Tang, C.C. TCC46* (HKU)	16 May 2011	KM818563	KM818628	KM818644	KM818705	KM818900	KM818953	KM818989	KM818812	KM818764
*Goniothalamus woodii* Merr. ex Mat-Salleh	Borneo	*Shea, G. SAN 75202* (L)	18 Mar 1972	KM818564	KM818629	KM818668	KM818720	KM818862	KM818972	–	KM818824	KM818778
*Goniothalamus wrayi* King	Peninsular Malaysia	*Suppiah, T. FRI 28345* (L)	18 Jan 1979	KM818565	KM818630	KM818671	KM818721	KM818859	KM818957	–	KM818803	KM818779
*Goniothalamus wynaadensis* Bedd.	India	*Kramer, K.U. 6248* (L)	17 Dec 1977	KM818566	KM818631	–	KM818697	KM818863	KM818970	KM818991	KM818816	KM818768
*Neostenanthera myristicifolia* (Oliv.) Exell	Gabon	*Wieringa, J.J. et al. 3566* (WAG)		EF179271	AY743486	EF179306	AY743448	AY743467	EF179348	–	AY743448	–
